# Effects of Dementia Care Mapping on job satisfaction and caring skills of staff caring for older people with intellectual disabilities: A quasi‐experimental study

**DOI:** 10.1111/jar.12615

**Published:** 2019-05-13

**Authors:** Feija D. Schaap, Evelyn J. Finnema, Roy E. Stewart, Geke J. Dijkstra, Sijmen A. Reijneveld

**Affiliations:** ^1^ Research Group Living, Wellbeing and Care for Older People NHL University of Applied Sciences Leeuwarden The Netherlands; ^2^ Department of Health Sciences, Community & Occupational Medicine, University Medical Center Groningen University of Groningen Groningen The Netherlands; ^3^ Department of Health Sciences, Applied Health Research, University Medical Center Groningen University of Groningen Groningen The Netherlands

**Keywords:** dementia, dementia care mapping, effect, intellectual disability, job satisfaction, person‐centred care

## Abstract

**Background:**

The ageing of people with intellectual disabilities, involving consequences like dementia, creates a need for methods to support care staff. One promising method is Dementia Care Mapping (DCM). This study examined the effect of DCM on job satisfaction and care skills of ID‐care staff.

**Methods:**

We performed a quasi‐experimental study in 23 group homes for older people with intellectual disabilities in the Netherlands. Among staff, we assessed job satisfaction and care skills as primary outcomes and work experience measures as secondary outcomes (*N* = 227).

**Results:**

Dementia Care Mapping achieved no significantly better effect than care as usual (CAU) for primary outcomes on job satisfaction (MWSS‐HC) and working skills (P‐CAT). Effect sizes varied from −0.18 to −0.66. We also found no differences for any of the secondary outcomes.

**Conclusion:**

Dementia Care Mapping does not increase job satisfaction and care skills of staff caring for older people with intellectual disabilities. This result differs from previous findings and deserves further study.

## 1 BACKGROUND

The ageing of the population with intellectual disabilities is accompanied by an increased risk of dementia and creates a need for methods to support ID‐care staff in their daily work (Cleary & Doodey, 2016; Duggan, Lewis, & Morgan, 1996). Dementia leads to a wide range of changes in memory, functional capacity, communication, neurology, personality and behaviour, and can result in agitation, resistance, depression and apathy (Ball, Holand, Treppner, Watson, & Huppert, 2008; Cleary & Doody, 2017; Emerson, 2001; Sheehan, Ali, & Hassiotis, 2014). These responses have a great impact on the lives of the people with intellectual disabilities, their housemates and their care staff (Cooper, 1997; Janicki & Keller, 2012; Shooshtari, Martens, Burchill, Dik, & Naghipur, 2011; Strydom, Chan, King, Hassiotis, & Livingston, 2013; Webber, Bowers, & McKenzie‐Green, 2010). This a potential challenge to ID‐care staff, who often lack the knowledge and skills to adapt to the changing behaviour, responses and needs of their clients (Cleary & Doodey, 2016; Iacono, Bigby, Carling‐Jenkins, & Torr, 2014; Janicki, 2011; Myrbakk & von Tetzchner, 2008). This lack can lead to low job satisfaction, stress and burnout (Ineland, Sauer, & Molin, 2017; Langdon, 2007; Mills & Rose, 2011; Pruijssers et al., 2015; Rose, Mills, Silva, & Thompson, 2013; Vassos & Nankervis, 2012), and creates a strong need for an evidence‐based method to help professionals to appropriately support their ageing clients (Duggan et al., 1996; Iacono et al., 2014; Watchman, 2008; Wilkinson, Kerr, & Cunningham, 2005). Such methods can be derived partly from standard geriatric and dementia care, as, for example, the use of person‐centred approaches (Bickenbach et al., 2012; Campens et al., 2017; Hales, Ross, & Ryan, 2006).

Person‐centred methods have been associated with improved quality of care, resulting in (psychosocial) benefits for both the people with dementia and their care staff (Brown et al., 2016; Brownie & Nancarrow, 2013; Edvardsson, Sandman, & Borell, 2014; Kuiper, Dijkstra, Tuinstra, & Groothoff, 2009; Rokstad et al., 2013; Willemse et al., 2015). Person‐centred care includes valuing the person, using an individual approach that acknowledges the uniqueness of the person, making an effort to understand the world from the perspective of the person and providing a supportive social environment (VIPS; Brooker, Woolley, & Lee, 2007). Organizations which perform well in person‐centred care create more productive interactions between healthcare professionals and clients, leading to a decrease in negative responsive behaviour of clients (Van der Meer, Nieboer, Finkenflügel, & Cramm, 2017; Willems, Embregts, Bosman, & Hendriks, 2014). Furthermore, person‐centred methods have been shown to improve quality of care, thereby increasing the well‐being of older people with intellectual disabilities, and contributing to job satisfaction of care staff (Brown et al., 2016; Cleary & Doody, 2017; De Vreese et al., 2012; Van der Meer et al., 2017).

One such person‐centred method is Dementia Care Mapping (DCM). This method supports dementia care staff working in psychogeriatric nursing homes, to improve the quality and effectiveness of care for people with dementia (see Box ) (Kitwood, 1992). DCM is an intensive observational tool used within a cycle of practice development in care settings, and simultaneously an approach to achieve and embed person‐centred care for people with dementia (Surr et al., 2016). DCM prepares staff to take the perspective of the person with dementia in assessing the quality of the care the staff provide. It is designed to empower teams to engage in evidence‐based critical reflection in order to improve quality of care at the individual level (clients and care staff), group level (staff and multidisciplinary teams) and management level, claiming that such improvement leads to higher job satisfaction of care staff (Kitwood, 1992; Van de Ven et al., 2013). A number of studies on DCM in nursing home settings found that it leads to less agitation, affective problems and verbal agitation in people with dementia (Chenoweth et al., 2009; Kuiper et al., 2009), and that it benefits for staff by improving caring skills, leading to increased job satisfaction, which includes a direction of decreased stress and risk of burnout (Barbosa, Lord, Blighe, & Mountain, 2017; Jeon et al., 2012; Kuiper et al., 2009). Jeon et al. (2012) and Van de Ven et al. (2013) found over time a greater decline in stress and emotional exhaustion, fewer negative emotional reactions (such as nervousness) and more positive reactions (such as optimism), among staff in the DCM group than in the control group, although this was not a significant difference (Jeon et al., 2012; Van de Ven et al., 2013). Van de Ven et al. (2013) also found that, over time, staff in the DCM group were slightly more satisfied with their job than the control group, although this was not significant either (Van de Ven et al., 2013).

Box 1 **Structure and contents of Dementia Care Mapping**

**Figure 1:**
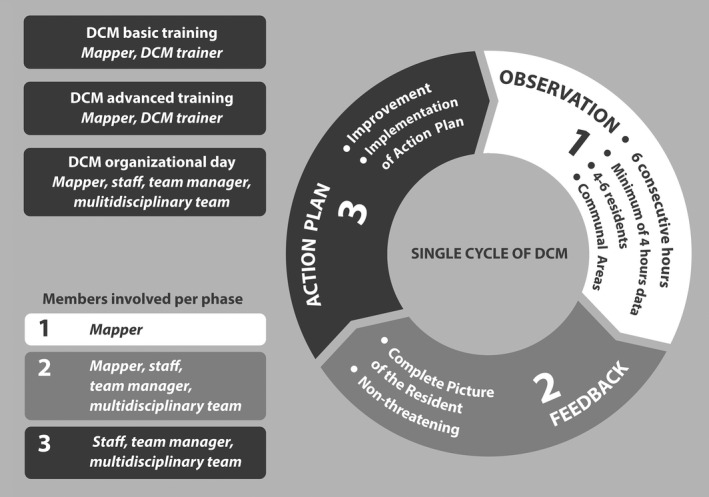
Dementia Care Mapping intervention components and cycle (based on: Van de Ven (2014))

1 Dementia Care Mapping (DCM) is an intervention developed by the Dementia Research Group at Bradford University, to improve the quality and effectiveness of care from the perspective of people with dementia (Brooker & Surr, 2005). It is based on Kitwood's social‐psychological theory of personhood in dementia (Kitwood, 1992). DCM was designed as observational tool to develop person‐centred care of people with dementia in nursing homes (Van de Ven et al., 2013). Person‐centred dementia care can be specified as: valuing people with dementia (V); using an individual approach that recognizes the uniqueness of the person (I); making an effort to understand the world from the perspective of the person (P); and providing a supportive social environment (S) (VIPS; Brooker et al., 2007). DCM has three main components (see also Figure 1):

A: Mappers’ training in DCM

A staff member receives training to become a certified DCM mapper. A basic DCM mappers’ course includes 4 days of basic concepts and skills. To participate in research, a mapper must achieve the level of advanced mapper. Required for this is a 3‐day course focused on the background and theory of DCM and person‐centred care. An advanced DCM mapper can observe (map) care with an inter‐reliability score of ≥0.8, report the observation, provide feedback and instruct staff in drawing up action plans (Van de Ven et al., 2013).

B: Organizational introductory briefing

Before the mapping (systematic observation of the actual care) takes place, the staff of a group home receives a short introduction (2 hr). This introduction explains the basic principles of DCM and person‐centred care to ensure endorsement and appropriate implementation (Van de Ven et al., 2013).

C: DCM cycle: observations‐feedback‐action plan

The introductory DCM organizational briefing day is followed by a DCM cycle, which consists of:

1. Observation, analysis and report*.* A mapper observes four to six residents in communal areas for 4–6 consecutive hours. Each 5‐min time frame a code is noted to record what happened to each resident and the associated behaviour of the staff. The DCM coding protocol contains 23 behavioural category codes (BCCs), well/ill‐being (WIB) values, personal detractions (PDs) and personal enhancers (PEs) in staff–client interactions (Brooker & Surr, 2005).
2. Feedback. The results of the mapping are communicated to the staff. The purpose of this feedback is to observe residents’ behaviour in the context of both their lives and the care (Brooker & Surr, 2005). Feedback is presented in a non‐threatening way and is intended to raise staff awareness of their own and residents’ behaviour, thereby motivating them to improve their competences, performance and interactions (Van de Ven et al., 2013).
3. Action plans*.* Based on the feedback, the staff draws up action plans to improve care at individual and group levels. Action plans are tools to implement in daily practice the principles of person‐centred care.

In ID‐care DCM has as yet been little used, but has been found promising in providing good care for older people with intellectual disabilities—whether or not with dementia (Finnamore & Lord, 2007; Jaycock, Persaud, & Johnson, 2006; Persaud & Jaycock, 2001; Schaap, Dijkstra, Finnema, & Reijneveld, 2018). DCM was shown to be feasible for people with intellectual disabilities, with and without dementia, after tailoring case histories and examples to ID‐care, but without altering the core DCM‐principles and DCM‐codes (Schaap, Fokkens, Dijkstra, Reijneveld, & Finnema, 2018; Schaap, Dijkstra, et al., 2018). Nevertheless, evidence on its effectiveness is lacking (Schaap, Fokkens, et al., 2018; Schaap, Dijkstra, et al., 2018). The aim of this study was therefore to examine the effect of DCM on the job satisfaction and (person‐centred) working skills of staff caring for older clients with intellectual disabilities.

## 2 METHODS

### 2.1 Study design

Between November 2014 and April 2016, we performed a quasi‐experimental study comparing DCM with care as usual, using a baseline measurement and follow‐up measurements after 7 and 14 months.

### 2.2 Study setting and participants

We performed a two‐stage sampling, first sampling ID‐care organizations and next assigning homes per organization to either the DCM or the control condition. First, we approached six ID‐care organizations with group homes for older clients in the north of the Netherlands; all were willing to participate (100%). Second, each organization provided four group homes for the study. In a group home, a small number (range 4–12) of older people with intellectual disabilities live together and receive care, support and supervision by care staff. In these group homes, 55% of the clients had a diagnosis or strong suspicion of dementia. We collected data from all care staff involved in the direct care process in these homes, that is those who supported residents in all aspects of day‐to‐day life, including activities of daily living (ADL) and day care activities.

Inclusion criteria for the group homes regarded: the possibility to observe four people simultaneously in a public area for at least 2 consecutive hours, the presence of at least three older people with (a strong suspicion of) dementia and a stable team without an anticipated reorganization. We balanced the representation of organizations between the control and intervention groups by allocating, of the four group homes per organization, two homes to the intervention group and two homes to the control group. Allocation of a group home to the intervention or control group depended on the geographical distance between the mapper and the home, as well as sufficient geographic distance between control and intervention group homes to prevent contamination.

### 2.3 Intervention

The intervention consisted of two applications of a full DCM cycle (Box) per group home, using the DCM‐in‐ID version, with an interval of 6 months. In this cycle, the managers of each participating group home first selected a staff member with the required competences to become a “DCM mapper” (i.e., a trained observer) (see Box). DCM Netherlands trained these twelve staff members to an advanced DCM‐level, meaning that they were able to carry out DCM: to observe (map) with an inter‐rater reliability agreement of at least ≥0.8, report, provide feedback, and instruct and support in drawing up action plans (Van de Ven et al., 2013). Second, a DCM trainer and a mapper jointly provided the DCM organizational introductory briefing in the group home. Third, the mappers carried out two full DCM cycles, consisting of a structured observation, feedback and action planning. A full cycle includes the following steps. First, the mappers observe four clients for 4–6 hr in communal areas in a group home. The results of the observation are reported to the staff, in order to help them understand clients’ behaviour in the context of their lives and their care (Brooker & Surr, 2005). The feedback is intended to increase insights and awareness of staff as to their own and clients’ behaviour, as well as staff–client interactions (Van de Ven et al., 2013). A researcher observed the feedback sessions, for the evaluation of the process of DCM. Based on the feedback, the staff made action plans to improve care at individual and group levels, by improving their own competences, performance and interactions. The application of DCM was in close cooperation with the DCM trainers, to guarantee accurate implementation; the DCM trainers checked the reports and jointly provided the feedback with the DCM‐in‐ID mappers. The action plans were sent to the mappers and DCM Netherlands. To maintain independence and to avoid interpretation bias due to familiarity with habits, clients and colleagues, the mappers carried out DCM in each other's organizations. More detailed information on the DCM procedures is provided in the Box.

The DCM trainers strictly monitored the intervention and supported the newly trained mappers in carrying out DCM following the DCM‐in‐ID implementation protocol (Bradford Dementia Group, 2014), which includes a description of all DCM pre‐conditions and of every step needed to implement DCM in ID‐care (Bradford Dementia Group, 2014). This protocol ensured that DCM was implemented and applied similarly in each group home and enabled a comparison of the group homes, even though these differed in (staff‐team) size, number of residents, culture and approach.

### 2.4 Control condition

The control condition was care as usual (CAU): continuous care with use of regular services (support in all aspects of day‐to‐day life, including activities of daily living [ADL] and day care activities) but no DCM. After the study period, the control group homes were offered a DCM‐training day upon which DCM could be implemented.

### 2.5 Procedure

We collected data from all care staff at three time points: at baseline, and after 7 and 14 months (i.e., 3 months after each application of DCM in the intervention group). Staff could choose to fill in the questionnaire on online or on paper. Personal details were anonymized by giving each staff member an identification number.

### 2.6 Outcome measures

Primary outcome measures were self‐reported job satisfaction, person‐centred care skills and quality of dementia care. We measured job satisfaction of care staff with the *Maastricht Work Satisfaction Scale in Health Care* (MWSS‐HC). This is a validated and reliable questionnaire which relates best to previous studies of care staff in various settings. It has also been used in studies of DCM in nursing home settings (Kuiper et al., 2009; Van de Ven, 2014). The MWSS‐HC is a 21‐item questionnaire using a five‐point Likert scale response format, from “very dissatisfied” (1) to “very satisfied” (5). All items relate to the job satisfaction of healthcare workers, divided into seven subscales of three items each, regarding satisfaction with: the manager, promotion possibilities, quality of care, opportunity to grow, contact with colleagues, contact with clients and clarity of the task. Scores are the mean of all items, with higher scores denoting greater job satisfaction. Table 1 provides further (psychometric) details on this questionnaire.

**Table 1:** Properties of used outcome measures

Name	Internal consistency	Inter‐rater reliability	Test–retest reliability	Mean (*SD*)	Validated for care staff	Nr questions/ answers	Separate use of subscales	Responsive to change	Previous use in DCM research	Domains/subscales
MWSS‐HC^a^, ^b^	*α* ≥ 0.84	*r* ≥ 0.50	N/A	3.43 (0.39)	✓	21/5	✓	✓	✓	Job satisfaction Subscales: satisfaction with The manager Promotion possibilities Quality of care Opportunity to grow Contact with colleagues Contact with clients Clarity of task
P‐CAT^a^, ^c^	*α* ≥ 0.83	*r* ≥ 0.82	*r* ≥ 0.82	2.53 (0.54)	✓	13/5	✓	✓	✓	Person‐centred care Subscales: Extent of personalizing care Amount of organizational support Degree of environmental accessibility
SCIDS^d^, ^e^	*α* ≥ 0.91	*r* ≥ 0.74	*r* ≥ 0.73	55.63 (7.48)	✓	17/4	✓	✓	✓	Sense of confidence in dementia care Subscales: Professionalism Building relationships Care challenges Sustaining personhood
SISE^d^, ^f^	N/A	*r* ≥ 0.88	*r* ≥ 0.75	3.5 (1.1)	✓	1/5		✓		
UWES‐9^d^, ^g^	*α* ≥ 0.93	*r* ≥ 0.65	*r* ≥ 0.46	3.74 (1.17)	✓	9/7	✓	✓		Subscales: Vitality Dedication Absorption
Dedication^d^, ^g^	*α* ≥ 0.92	*r* ≥ 0.65	*r* ≥ 0.69	3.91 (1.31)	✓	5/7				
Professional efficacy^d^, ^h^	*α* ≥ 0.83	*r* ≥ 0.90	*r* ≥ 0.86	4.87 (1.61)	✓	6/7		✓	✓	Professional efficacy
Work Perception^d^, ^i^	*α* ≥ 0.77	N/A	*r* ≥ 0.52	3.65 (1.04)	✓	3/5		✓		Work perception
VIPS^d^, ^j^	N/A	N/A	N/A	N/A		20/5	✓	✓		Used subscales (partly): Quality assurance Communication Empathy and acceptable risk Challenging behaviour as communication Recognizing and responding to change Inclusion Validation Warmth

a Primary outcome.

b Landeweerd, Boumans and Nissen (1996) and Rövekamp, Schoone‐Harmsen, and Oorthuizen (2009).

c Edvardsson, Fetherstonhaugh, Nay, and Gibson (2010).

d Secondary outcome.

e Schepers, Orrell, Shanahan, and Spector (2012).

f Robins, Hendin, and Trzesniewski (2001).

Internal consistency cannot be computed for a single‐item scale.

g Schaufeli and Bakker (2004a, 2004b).

h Subscale of UBOS/Maslach Burnout Scale: Schaufeli and Van Dierendonck (2000), Schaufeli, Bakker, Hoogduin, Schaap, and Kladler (2001) and Schutte, Toppinen, Kalimo, and Schaufeli (2000).

i De Jonge (1995) and De Jonge et al. (1995).

j Brooker (2011) Derived from: care fit for vips assessment tool: https://www.carefitforvips.co.uk/

We assessed person‐centred care skills and quality of dementia care, first measuring the level of the provided person‐centred care with the *Person‐Centred Care Assessment Tool* (P‐Cat; Edvardsson, Fetherstonhaugh, Nay, & Gibson, 2010), and second, with the *Sense of Competence in Dementia Care Staff Scale* (SCIDS; Schepers, Orrell, Shanahan, & Spector, 2012). The P‐CAT is an assessment scale whereby care staff can rate to what extent care is person‐centred. It is a validated scale, consisting of 13 items formulated as statements about the presence of person‐centredness in the group home (see Table 1). A five‐point scale ranging from 1 (disagree completely) to 5 (agree completely) is used for scoring. Items 8–12 are negatively worded, and the responses have to be reversed before analysis. The three subscales focused on personalizing care (seven items), organizational support (four items) and environmental accessibility (two items). The scores are the means of all items; higher scores indicate more person‐centred care in the group home. The SCIDS measures the sense of competence of care staff in dementia care. This is a validated questionnaire containing 17 items with a 4‐point Likert scale (see Table 1). All items are scored from 1 (not at all) to 4 (very much). Higher scores denote a greater level of sense of confidence. Scores are added up for items from 1 to 17 for the overall SCIDS score; higher scores indicate a higher level of confidence in dementia care. Subscales include professionalism (five items), building relationships (four items), care challenges (four items) and sustaining personhood (four items). We translated the SCIDS using a standard forward–backward method (Maneesriwongul & Dixon, 2004; Sousa & Rojjanasrirat, 2011). Two independent translations into Dutch (by two authors) were combined into a single version. A native English speaker, fluent in Dutch and with a medical background, translated this provisional Dutch version back into English. In case of deviations from the original English version, the Dutch translation was revised. This occurred in only a few cases, as the back translation was found to be nearly identical to the source text.

Secondary outcome measures regarded possible explanatory variables for job satisfaction and care skills, being: self‐reported self‐esteem, professional efficacy, commitment to work, work perception and provision of person‐centred care. We measured self‐esteem with the *single‐item self‐esteem scale* (SISE), a single item on a 5‐point Likert scale (Robins, Hendin, & Trzesniewski, 2001). The wording of the SISE is “Please indicate to what extent the following statement applies to you: *I have high self‐esteem.*” In various studies, the SISE was shown to be a reliable and valid instrument for measuring global self‐esteem (Bleidorn et al., 2016; Brailovskaia & Margraf, 2018; Erdle, Irwing, Rushton, & Park, 2010; Kırcaburun et al., 2018). The SISE was also translated according to the forward–backward method. We assessed commitment to work with the validated *Utrecht Commitment Scale* (UWES‐9; see Table 1). Its items are scored on a 7‐point Likert scale ranging from 0 (never) to 6 (always). The subscales vitality, dedication and absorption all contained three items. Scores are the mean of all items, and higher scores indicate a higher commitment to work. To gain deeper insight into the dedication of ID‐care staff, we added two items from the *dedication* subscale of the UWES‐15 (Schaufeli & Bakker, 2004a, 2004b)*.*

We assessed professional efficacy using the subscale “professional efficacy” from the *Utrecht Burn Out Scale* (UBOS—the Dutch equivalent of the *Maslach Burnout Inventory*; Schaufeli & Van Dierendonck, 2001; Schutte, Toppinen, Kalimo, & Schaufeli, 2000). We chose to use this subscale exclusively because its contents fitted the objectives of DCM, in contrast to the other parts of this measure. Professional efficacy was measured using a 7‐point Likert scale from 0 (never) to 6 (always). Its score is the mean of all items, higher scores denoting a higher professional efficacy. We measured work perception with the Work Perception scale, which contained questions regarding pleasure, contentedness and feelings regarding work (De Jonge, Boumans, Landeweerd, & Nijhuis, 1995). This is a three‐item, five‐point Likert scale from 1 (disagree completely) to 5 (agree completely). The mean of the score indicates the work perception of the staff member, with higher scores indicating a more positive work perception (see also Table 1). Lastly, we measured provision of person‐centred care provided by staff, using questions from the *Care fit for VIPS* assessment tool. This tool is based on principles for this type of care, as specified by Brooker (Brooker, 2011; Røsvik, Brooker, Mjorud, & Kirkevold, 2013), aspects which were not covered by the other questionnaires. We selected questions to measure change in time regarding this care. These questions were translated following the forward–backward method.

### 2.7 Sample size

We determined sample size based on the MWSS‐HC as primary outcome. To measure an effect size of 0.5 (i.e., a 0.2 point increase in the MWSS‐HC; Landeweerd, Boumans, & Nissen, 1996; Van de Ven et al., 2013), given a mean of 3.50 and a standard deviation (*SD*) of 0.40, at *α* = 0.05 (two‐sided) and power = 80% (Cohen, 1988), we needed twelve staff in each group (intervention group and control group). With adjustment for an estimated “loss to follow‐up” of 25%, we needed to include 2 × 16 staff in the study.

### 2.8 Data analysis and reporting

First, we described the flow of participants. Second, we assessed the baseline characteristics of the staff in each research group. The differences between the two groups were tested using Pearson chi‐square tests for categorical variables and one‐way analysis of variance (ANOVA) for continuous variables. Third, we compared the differences in change in time between the DCM and the CAU groups. We assessed the effects of DCM using intention to treat (ITT) analyses after the first DCM cycle (T0 to T1) and after the second DCM cycle (T0 to T2); all staff were analysed regardless of whether or not they had completed the intervention and any post‐intervention questionnaire. For analysis, we used multilevel mixed‐effect model techniques in which the time points were the first level (L1), the care staff the second (L2) and the group homes wherein care staff are nested the third (L3). We performed analyses using the unconditional means model (Singer & Willett, 2003). For each outcome, we calculated effect sizes for the differences in change between both groups.

We repeated these analyses with adjustment for covariates seen to have a significant influence on the intercept in the conditional means model, to examine whether this led to a major change in the outcomes. These covariates regarded age, gender, whether staff had been trained in person‐centred care and the number of years of experience in the current group home. We further adjusted for the percentages at group home level of people with profound and severe intellectual disabilities, and for the percentage of people with a diagnosis of dementia.

Finally, we performed a complete case analysis for the T1‐T0 and T2‐T0 comparisons. As an additional analysis, we repeated these analyses, excluding subscales that DCM not could influence. These were three subscales of MWSS‐HC: “being satisfied with the manager,” “the possibilities to gain promotion” and “growth in the organisation.” This also applies to one subscale of P‐CAT, “environmental accessibility.”

Analyses were performed using IBM SPSS Statistics version 25.0 and MLWin version 2.35. Our report followed the CONSORT checklist (Schulz, Altman, & Moher, 2010).

### 2.9 Ethical permission

The Medical Ethical Committee of the University Medical Center Groningen considered approval unnecessary (decision M13.146536), because DCM is an intervention aimed at staff. Written informed consent was obtained from representatives of the people with intellectual disabilities involved in the study. The trial has been registered in the Dutch Trial Register, number NTR2630.

## 3 RESULTS

### 3.1 Participant flow

**Figure 2:**
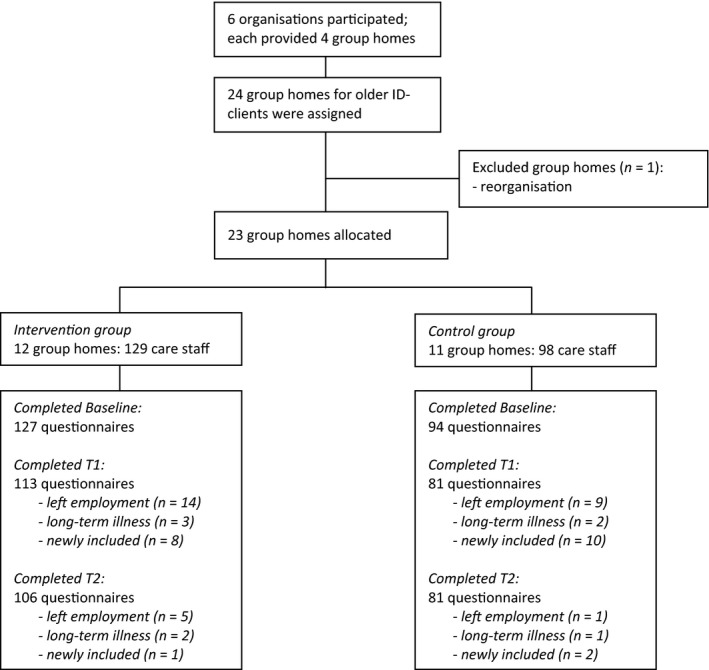
Flowchart detailing numbers of group homes and staff members by condition

Figure 2 shows the flow of staff through the study. We collected data from all staff involved in each group home. In total, 221 filled in the baseline measurement, 127 in the intervention group and 94 in the control group. Overall, 136 staff in the intervention group and 106 staff in the control group completed a questionnaire on at least one time point (Figure 2). For complete case analysis, we included 92 staff in the intervention group and 62 in the control group.

### 3.2 Background characteristics

Staff in the intervention and control groups did not differ regarding any background characteristics (Table 2). At group home level, the percentage of clients diagnosed with dementia in the DCM group was significantly higher than in the CAU group (Table 2).

**Table 2:** Background characteristics staff and group homes

	DCM	CAU	*p*‐value
Staff			
*N*	127	94	
Mean age in years (*SD*)	45 (12.4)	44 (12.1)	0.68
Female (%)	90	90	0.50
Education			0.74
Elementary/secondary education (%)	9	9	
Secondary vocational education (%)	80	77	
Higher professional education (%)	11	13	
Position			0.36
Daily care professional (%)	63	69	
Senior/coordinating care professional/personal coach (%)	32	30	
Permanent employment (%)	90	93	0.81
Hours/week (mean)	23	24	0.84
Experience			
>11 years in ID‐care (%)	69	61	0.29
>11 years in current group home (%)	32	24	0.59
Experienced with person‐centred care (%)	84	79	0.70
Education of older people with intellectual disabilities (%)	76	69	0.23
Psychosocial approach/method in group home (%)	71	71	0.92
Group homes			
*N*	113	111	
Mean age in years (*SD*)	67 (11.3)	65 (12.4)	0.38
Female (%)	43	56	0.05
Mean years in current organization (*SD*)	31 (15.6)	27 (13.8)	0.05
Mean years in current location (*SD*)	8 (5.9)	10 (8.2)	0.033*
Clients with degree of disability			0.004*
Mild (%)	21	31	
Moderate (%)	49	56	
Severe/Profound (%)	31	13	
Clients with dementia			0.003*
Diagnosed (%)	35	17	
Suspicion/Signs of (%)	29	29	

* Significant difference between DCM and CAU group (*p *= <0.05).

### 3.3 Effects on primary and secondary outcomes

Table 3 presents the effects of DCM compared to CAU. Between groups, we found no differences in change regarding any of the primary outcomes (MWSS‐HC, P‐CAT and SCIDS), between T0 and T1, and between T0 and T2. Effect sizes varied from −0.18 to −0.47 for T0‐T1 and from −0.30 to −0.66 for T0 to T2. Regarding the secondary outcomes, we also found no differences between T0 and T1 and T0 and T2. Effect sizes varied from 0.08 to −0.29 for T0‐T1 and from −0.03 to −0.17 for T0 to T2.

**Table 3:** Raw means at T0, T1 and T2, based on intention to treat analyses with mixed multilevel models (*n* = 227)

Outcome	Group	T0 (Baseline)		T1 (3 months after 1st DCM cycle)		Difference in improvement T0 to T1 between DCM and CAU			T2 (3 months after 2nd DCM Cycle)		Difference in improvement T0 to T2 between DCM and CAU		
		Mean^a^	*SD*	Mean^a^	*SD*	Dif^b^	*p*‐value	Effect size	Mean^a^	*SD*	Dif^b^	*p*‐value	Effect size
MWSS‐HC	DCM	3.88	0.40	3.86	0.35	−0.07	0.67	−0.18	3.80	0.37	−0.11	0.52	−0.30
	CAU	3.87	0.37	3.91	0.33				3.90	0.38			
P‐CAT	DCM	3.85	0.46	3.69	0.42	−0.21	0.48	−0.47	3.66	0.35	−0.29	0.42	−0.66
	CAU	3.77	0.48	3.83	0.45				3.88	0.44			
SCIDS	DCM	52.53	8.35	53.89	7.36	1.87	0.55	0.24	53.41	7.75	−0.23	0.10	−0.03
	CAU	53.68	7.55	53.17	7.38				54.79	6.74			
SISE	DCM	4.16	0.67	4.15	0.60	−0.19	0.12	−0.29	4.18	0.66	−0.06	0.33	−0.10
	CAU	4.00	0.69	4.19	0.71				4.09	0.60			
UBES9	DCM	5.72	0.90	5.68	0.85	0.16	0.21	0.18	5.65	0.84	0.11	0.12	0.13
	CAU	5.70	0.87	5.49	0.87				5.52	0.84			
Professional Efficacy^c^	DCM	5.70	0.84	5.82	0.79	0.23	0.89	0.28	5.75	0.76	0.13	0.31	0.16
	CAU	5.79	0.78	5.68	0.83				5.71	0.74			
Work Perception^c^	DCM	0.00	0.94	−0.03	0.88	−0.09	0.67	−0.10	−0.06	0.93	−0.15	0.98	−0.17
	CAU	−0.02	0.76	0.04	0.86				0.07	0.82			
VIPS^c^	DCM	0.00	0.59	0.02	0.53	0.05	0.84	0.08	−0.01	0.62	−0.02	0.63	−0.04
	CAU	0.00	0.58	−0.03	0.60				0.01	0.60			

a Raw mean scores on the different outcome measurements.

b Based on mixed model techniques, expressing differences in change between DCM and CAU in outcomes.

Effect size (Cohen's d).

Primary outcome.

c Secondary outcome.

Based on Z‐scores; DCM: intervention group; CAU: control group—care as usual.

Adjustment for covariates did not notably affect findings; effect sizes on the primary outcomes with adjustment for covariates varied from −0.16 to −0.30 for T0 to T1 and from −0.05 to −0.52 for T0 to T2, and for the secondary from 0.07 to −0.30 for T0 to T1 and from −0.04 to −0.16 for T0 to T2. The complete case analysis yielded similar findings. Additional analyses with exclusion of less relevant subscales of MWSS‐HC and P‐Cat also did not affect findings.

## 4 DISCUSSION

The lack of effect of DCM on job satisfaction and working skills seems to contradict promising findings in earlier studies on DCM in ID‐care (Finnamore & Lord, 2007; Jaycock et al., 2006; Schaap, Fokkens, et al., 2018; Schaap, Dijkstra, et al., 2018). This contrast between our study and previous ones may be explained in several ways. First, staff scored high at baseline in all outcomes, except for competence in dementia, leading to a ceiling effect in measuring effects. Regarding job satisfaction (MWSS‐HC), the participants scored one standard deviation higher than the norm population (Landeweerd et al., 1996). Also regarding person‐centred working skills (P‐Cat) and the secondary measures self‐esteem, professional efficacy and commitment to work, the participants scored high at baseline compared to the norms (De Jonge et al., 1995; Edvardsson et al., 2010; Hastings, Horne, & Mitchell, 2004; Robins et al., 2001; Schaufeli & Van Dierendonck, 2001; Schaufeli & Bakker, 2004b; Schutte et al., 2000). This may be because secondary vocational trained professionals are less accustomed to reflect on their own job performance and may base their answers on a (high) self‐imposed standard (Dunning, Johnson, Ehrlinger, & Kruger, 2003; Kruger & Dunning, 2009). Moreover, our finding of high engagement, involvement and dedication on the part of ID‐care staff aligns with findings of previous studies among care professionals who have built long‐term caring relationships with their clients. This largely differs from many other (dementia) care settings (Bekkema, de Veer, Hertogh, & Francke, 2015; Finkelstein, Bachner, Greenberger, Brooks, & Tenenbaum, 2018; Iacono et al., 2014; Wagemans, 2013). Such high self‐esteem, and commitment to work may cause overestimation of their performance possibilities, reflected in taking on overly demanding responsibilities and refusing to admit mistakes in their jobs (Baumeister, Heatherton, & Tice, 1993; Donaldson & Grant‐Vallone, 2002; Holtz & Gnambs, 2017; Janssen & Van der Vegt, 2011; Murray, 2005). Moreover, an increased level of confidence is not necessarily consistent with an increased level of knowledge (Leopold et al., 2005; Webber, Bowers, & Bigby, 2016).

Second, in our study DCM was carried out by ID‐care professionals newly trained in the intervention, which may have weakened the intervention. Previous research has stressed the importance of strict adherence to the DCM‐implementation protocol (Chenoweth et al., 2015; Rokstad, Vatne, Engedal, & Selbæk, 2015; Van de Ven et al., 2014). However, the strict monitoring of intervention fidelity in this study makes this explanation less likely (Schaap, Dijkstra, et al., 2018). Moreover, the two previous studies to assess the effect of DCM on dementia care staff both made use of experienced mappers, but offering either one (Jeon et al., 2012) or two DCM cycles with newly trained mappers (Van de Ven et al., 2013). None of them found significant effects on job satisfaction and care skills, but they found improvement of negative work experiences (Jeon et al., 2012; Van de Ven et al., 2013; Barbosa et al., 2017).

Third, DCM may simply not lead to better job satisfaction. As in previous studies, we have connected our outcome measures to the claim that DCM increases job satisfaction. Studies on DCM that aimed at dementia care staff found improved caring skills, leading to increased job satisfaction, which included a tendency of reduced stress, burnout and emotional exhaustion as well as less negative and more positive reactions to clients, although this was not significant (Barbosa et al., 2017). DCM may thus indirectly improve some negative work experiences but its effects may be too weak to improve job satisfaction. This applies even more to the paradigm‐shift towards person‐centred care in the entire organizational culture.

### 4.1 Strengths and limitations

Our study had a number of strengths. First, we used a version of DCM already adapted to ID‐care (Schaap, Fokkens, et al., 2018). Next, our study had a large sample size, participants from a wide range of organizations, an independent data collection, ample strategies to avoid contamination and bias, a comparable control group and a long follow‐up of 1 year with two follow‐up measurements. Furthermore, our study had low loss to follow‐up.

Nevertheless, we must also note limitations. First, by using self‐report questionnaires we relied fully on self‐report by staff; this may have led to information bias and a less accurate measurement of change. In our study, self‐reported scores at baseline were rather high and may have caused a ceiling effect, even though the outcome measures were valid and sensitive for this group. This ceiling effect may have limited the potential to measure the effects of DCM.

Second, the intervention and control groups differed regarding some background characteristics. These regarded a greater severity of the disability and a higher prevalence of dementia diagnoses in the DCM group. However, adjustment for these differences did not affect the findings. Third, the new ID‐mappers were trained using a not yet fully adapted version of ID‐care, although in a pilot this version had been shown to be adequate (Schaap, Fokkens, et al., 2018). Furthermore, we have accomplished integrity checks of the products of the observation, that is the reports and action plans, but not of the observation process itself. We thus cannot be fully sure of correct implementation of DCM, but the products at least had reached an adequate level. Moreover, a process analysis of the implementation of DCM in the group homes showed that this was in accordance with the DCM‐in‐ID protocol, and the fidelity to this protocol was strictly monitored and supported by DCM trainers (Schaap, Dijkstra, et al., 2018).

### 4.2 Implications

In this first implementation of DCM in ID‐care, we found no evidence that DCM increases job satisfaction, (dementia/person‐centred) working skills and knowledge of ID‐care staff, making it questionable whether DCM should be implemented to improve these issues. Yet prior and qualitative studies provided strong indications that person‐centred care, with methods such as DCM, does improve care by enhancing the knowledge and skills of ID‐care staff (Bertelli, Salerno, Rondini, & Salvador‐Carulla, 2017; Kendrick, 2011; Schaap, Fokkens, et al., 2018; Van der Meer et al., 2017). Further research is needed to elucidate this discrepancy, for example by in‐depth interviews with participating ID‐staff or direct observation, and by including more stressed staff to, for example a lower staff/resident ratio. The effects of DCM on outcomes of older people with intellectual disabilities, such as quality of life, should also be examined as this may provide more proximal measures. Moreover, different outcome measures that are more closely related to the intervention such as quality of care and quality of staff–client interactions should be included. Finally, a longer follow‐up period may be useful, as a transition to more person‐centred care may require more time than provided by the follow‐up of our study. The promising option of DCM in ID‐care thus deserves further study.

## 5 CONCLUSION

Contrary to previous studies that reported that DCM and person‐centred care provide (intellectual disability) staff greater knowledge and skills in providing dementia care, we found no evidence that DCM increases their job satisfaction and dementia‐ and person‐centred working skills. This discrepancy requires further study.

## CONFLICT OF INTEREST

The authors have no conflict of interest to declare.
